# Evaluation and Selection of Thermal Processing Conditions for Safety, Flavor Retention, and Shelf-Life Extension of Fermented Pickled Mustard Greens

**DOI:** 10.3390/molecules31132289

**Published:** 2026-07-01

**Authors:** Qiuming Chen, Shikang Chen, Junjie Tong, Zhaojun Wang, Maomao Zeng, Zhiyong He, Jie Chen

**Affiliations:** 1 State Key Laboratory of Food Science and Resources, Jiangnan University, Wuxi 214122, China; chenqm@jiangnan.edu.cn (Q.C.); 6240111181@stu.jiangnan.edu.cn (S.C.);; 2 School of Food Science and Technology, Jiangnan University, Wuxi 214122, China; 3 Collaborative Innovation Center of Food Safety and Quality Control, Jiangnan University, Wuxi 214122, China

**Keywords:** fermented pickled mustard greens, thermal treatment, thermal death kinetics, isothiocyanates, sensory quality, shelf-life prediction

## Abstract

To address post-acidification, microbial contamination, and quality deterioration in fermented pickled mustard greens after fermentation, this study systematically evaluated the effects of thermal treatment on quality preservation, selected a preferred thermal processing condition from three kinetically designed treatments, and predicted product shelf life. Based on heat penetration curves and the thermal death kinetics of the target heat-resistant microorganism, *Bacillus subtilis*, three thermal processing conditions were established: 75 °C for 64 min, 85 °C for 19 min, and 95 °C for 17 min. The D-value of *B. subtilis* spores at 85 °C was 1.37 min, and the corresponding thermal treatments were designed according to a 2D reduction principle. HS-SPME-GC-MS analysis identified 84 volatile compounds, with isothiocyanates representing key contributors to the characteristic pungent aroma of mustard-based pickles. Sensory evaluation showed that the 85 °C treatment group achieved the best observed balance among pungency, refreshing aroma, mellow flavor, color, texture, and overall acceptability, whereas excessive heating at 95 °C promoted isothiocyanate loss and texture deterioration. During accelerated storage, the selected treatment inhibited post-acidification, maintained nitrite at a low level (<1 mg/kg), and delayed microbial and sensory deterioration. Integrating physicochemical indices, microbial populations, and sensory scores, the theoretical shelf life under refrigeration at 4 °C was predicted to be 170 days using the Q10 model. These findings provide practical guidance for selecting thermal processing conditions that balance microbial safety, flavor retention, and shelf-life extension in industrial fermented pickled mustard greens.

## 1. Introduction

Fermented vegetables (such as Chinese paocai, Korean kimchi, and German sauerkraut) are highly favored by global consumers for their unique flavor, crisp texture, and rich content of dietary fiber, minerals, and probiotic metabolites [1]. During the natural or inoculated fermentation process of traditional pickles, the microbial community, dominated by lactic acid bacteria, converts carbohydrates into lactic acid, organic acids, and various volatile flavor compounds through complex biochemical reactions, which constitute the core quality characteristics of pickles [2]. However, after traditional pickles reach fermentation maturity, many active fermenting microorganisms, endogenous enzymes, and potential spoilage bacteria still exist in the system. If left untreated, the products are highly susceptible to quality deterioration during subsequent storage and distribution [3]. This deterioration is primarily manifested as “post-acidification” caused by the excessive metabolism of residual lactic acid bacteria, texture softening resulting from the enzymatic degradation of pectin, as well as gas production (package swelling) and flavor spoilage caused by the proliferation of miscellaneous bacteria such as yeast, enterobacteria, or *Bacillus* [4]. These issues not only severely shorten the shelf life of the products but also greatly restrict the large-scale and standardized development of the fermented vegetable industry.

To control post-ripening fermentation, inhibit spoilage microorganisms, and ensure food safety, implementing scientific sterilization treatments for fully fermented pickles is a necessary step for industrialized production [5]. In recent years, although non-thermal sterilization technologies such as high hydrostatic pressure, ultrasound treatment, and irradiation have shown potential in preserving the sensory and nutritional qualities of food [6], they are currently difficult to widely popularize in the large-scale factory production of pickles due to high equipment investment, limited processing throughput, and the potential to induce lipid oxidation [7]. In contrast, thermal processing remains the mainstream and preferred technology in the current food industry for controlling microbial risks and extending shelf life due to its mature technology, simple operation, low cost, and ease of engineering scale-up [8].

However, applying heat treatment presents a complex challenge regarding the overall quality of pickles. On one hand, insufficient thermal intensity (e.g., temperatures that are too low or processing times that are too short) fails to effectively inactivate highly stress-resistant spoilage microorganisms within the product [9]. The raw materials of fermented vegetables often carry *Bacillus* spp. originating from the soil; these microorganisms can form heat-resistant spores and represent a stubborn microbial group that causes the spoilage of acidic canned foods [10]. On the other hand, excessive heat treatment causes irreversible damage to the physicochemical properties and sensory quality of the pickles [11]. High temperatures not only induce non-enzymatic browning (such as the Maillard reaction), leading to a darker color, but also destroy the cell wall structure and degrade pectin and cellulose, causing the pickles to lose their core crispness [12]. More importantly, the characteristic flavor of pickles is extremely sensitive to heat. For example, the signature spicy and refreshing aroma of mustard-based pickles mainly originates from sulfur-containing compounds (especially isothiocyanates). High-temperature heating easily leads to the volatilization, degradation, or conversion of these key aroma components into off-flavors [13].

In fermented pickled mustard greens, isothiocyanates derived from glucosinolates are among the most important characteristic aroma-active compounds. They contribute the typical pungent, fresh, and mustard-like notes that distinguish mustard-based pickles from other fermented vegetables. Because these sulfur-containing volatiles are thermally sensitive, excessive heating may weaken the characteristic aroma and reduce consumer acceptability [12,14].

Currently, when formulating heat treatment sterilization processes, many pickle processing enterprises often rely on empirical parameters, lacking the theoretical support of systematic thermal inactivation kinetics and heat transfer profiles. Furthermore, there are few reports on the remodeling mechanisms of the complex volatile flavor profiles of pickles under different heat treatment intensities, especially the evolution patterns of characteristic flavor substances analyzed using high-throughput flavoromics technologies. How to accurately control thermal parameters to maximize the retention of the crispness and authentic flavor of pickles while ensuring the complete eradication of target heat-resistant indicator bacteria remains a key technical bottleneck that urgently needs to be resolved in the current pickle processing field.

Previous studies have reported that fermentation conditions, microbial succession, and thermal treatment can reshape the volatile profiles of fermented vegetables and glucosinolate-derived flavor compounds [12,14,15]. However, systematic studies connecting thermal inactivation design, volatile flavor retention, sensory quality, and shelf-life prediction in fermented pickled mustard greens remain limited.

Based on the above background, this study aims to evaluate and select, from three kinetically designed heat treatments, a preferred condition that balances safety and sensory quality, and to scientifically predict its shelf life. The specific research objectives include: (1) selecting the highly heat-resistant *Bacillus subtilis* as the target indicator bacterium to establish its thermal inactivation kinetic model (D-value and Z-value) in a simulated pickle system, and combining it with the heat transfer characteristics (heating curves) of real vacuum-packaged pickles to scientifically calculate heat treatment parameters that meet commercial sterility requirements; (2) comprehensively analyzing the effects of different heat treatment conditions on the composition and content of volatile flavor compounds in pickles using headspace solid-phase microextraction combined with gas chromatography–mass spectrometry (HS-SPME-GC-MS) coupled with multivariate statistical analysis (PCA and PLS-DA); (3) selecting the preferred flavor-preserving thermal treatment in combination with quantitative descriptive sensory evaluation; and (4) based on the selected treatment, introducing an accelerated shelf-life testing (ASLT) model to systematically monitor dynamic changes in physicochemical and microbiological indicators under different storage temperatures, thereby estimating the cold-chain shelf life of the product. This study is expected to provide a solid theoretical basis and engineering practice guidance for the high-quality industrial production, targeted flavor regulation, and shelf-life extension of traditional fermented vegetables.

## 2. Materials and Methods

### 2.1. Materials and Reagents

Fermented mature pickled mustard greens, prepared using a standardized fermentation process, were used in this study. The product was vacuum packaged after fermentation maturity and used as the model fermented pickled mustard greens matrix. The target indicator strain, *Bacillus subtilis* 168 (standard strain), was stored in the laboratory’s culture collection. Tryptone, yeast extract, sodium chloride, and agar powder were all purchased from Sangon Biotech Co., Ltd. (Shanghai, China).

### 2.2. Determination of Heat Transfer and Thermal Inactivation Kinetics

The fermented pickles were vacuum packaged in 200 g portions. A thermocouple probe was placed at the geometric center (the cold spot) of each package to monitor the temperature. The samples were then submerged in water baths set at 75 °C, 85 °C, and 95 °C, and the heating rate at the cold spot was recorded in real-time to provide a basis for calculating the sterilization parameters.

Following activation and shaking culture, *B. subtilis* was inoculated into a modified spore-inducing medium and cultured for 5–7 days until the sporulation rate exceeded 90%. The suspension was treated at 80 °C for 10 min to eliminate vegetative cells, washed, and then resuspended in a sterile simulated pickle matrix, with the initial concentration adjusted to approximately 10^8^ CFU/mL. The thermal inactivation kinetics were determined using the capillary tube method (0.5 mm inner diameter) to minimize heat transfer lag. The capillary tubes containing the spore suspension were immersed in water baths at different temperatures and rapidly cooled in an ice-water bath at specified time intervals. The number of surviving spores was determined using the standard plate count method, and the logarithmic value of surviving spores (log N) was calculated to plot the thermal inactivation curves and determine the D and Z values.

### 2.3. Heat Treatment and Quality Assessment

Based on the 2D reduction principle, the vacuum-packaged pickles were subjected to heat treatments at 75 °C for 64 min, 85 °C for 19 min, and 95 °C for 17 min, respectively. An untreated group was set as the control (CK). Following the heat treatment, the samples were rapidly cooled to room temperature for subsequent analysis.

The volatile compounds in pickled mustard greens under different heat treatments were analyzed using headspace solid-phase microextraction combined with gas chromatography–mass spectrometry (HS-SPME-GC-MS), according to the method of Xu et al. [16] with slight modifications. Briefly, 2 g of homogenized pickle sample was placed in a 20 mL headspace vial, and 1 μL of 2-methyl-3-heptanone in methanol (820.0 mg/L) was added as the internal standard. The vial was equilibrated in a 50 °C water bath for 7 min. A preconditioned SPME fiber (50/30 μm DVB/CAR/PDMS) was inserted into the vial headspace and exposed at 50 °C for 30 min. After extraction, the fiber was immediately inserted into the GC injection port and desorbed for 7 min in splitless mode. Analysis was performed using a Shimadzu GC/MS-QP2020NX system (Shimadzu, Kyoto, Japan) equipped with an SH-WAX capillary column (30 m × 0.25 mm × 0.25 μm; Shimadzu, Kyoto, Japan). The injection-port temperature was 250 °C, and high-purity helium was used as the carrier gas at 2 mL/min. The oven temperature was held at 40 °C for 3 min, increased to 100 °C at 6 °C/min, then increased to 230 °C at 10 °C/min and held for 5 min. The sample effluent was split at a 1:1 volume ratio between the mass spectrometer and the olfactory detector (ODE-2030, Shimadzu, Kyoto, Japan). The mass spectrometer was operated in full-scan mode (*m*/*z* 33–400) using electron-impact ionization at 70 eV; the ion-source and interface temperatures were 200 °C and 250 °C, respectively. For the global volatile profile analysis, the detected compounds were semi-quantified by the internal-standard method, assuming a relative response factor of 1. The semi-quantitative content of compound i was calculated as *C*_i_ = (*A*_i_/*A*_(_IS_)_) × (*C*_(_IS_)_ × *V*_(_IS_)_/*m*), where *A*_i_ and *A*_(_IS_)_ are the peak areas of compound i and 2-methyl-3-heptanone, respectively; *C*_(_IS_)_ is the internal-standard concentration (820.0 mg/L); *V*_(_IS_)_ is the added internal-standard volume (1 μL); and m is the sample mass (2 g). Under these conditions, *C*_i_ = (*A*_i_/*A*_(_IS_)_) × 410 μg/kg. The results therefore represent semi-quantitative concentrations expressed as 2-methyl-3-heptanone equivalents, rather than compound-specific absolute concentrations established using an authentic-standard calibration curve.

A trained sensory evaluation panel consisting of 12 members evaluated the samples. The panel included well-trained students and teachers with experience in fermented vegetable sensory evaluation, who were expected to have higher sensitivity to aroma, taste, and texture differences than untrained consumers. Before formal evaluation, panelists were trained using representative pickle samples to familiarize them with the attribute definitions and the 9-point scoring scale. The panelists scored the samples based on color attributes (browning degree and color brightness), texture attributes (crispness and hardness), taste attributes (acidity and overall acceptability), and flavor attributes (spiciness, refreshing aroma, mellow aroma, herbal, cucumber, and floral notes). The final score for each attribute was the average of all evaluators, and the results were presented in a radar chart.

### 2.4. Accelerated Storage and Shelf-Life Prediction

Pickles treated under the selected heat treatment condition were stored at 4 °C, 30 °C, and 40 °C for accelerated shelf-life testing. Samples were collected every 7 days. Samples stored at 4 °C and 30 °C were monitored for 35 days, whereas samples stored at 40 °C were monitored for 21 days because obvious spoilage and sensory rejection occurred thereafter.

For physicochemical analysis, 10 g of pickle sample was mixed with 90 mL of deionized water and homogenized for 2 min. The pH was measured at room temperature using an FE28-Standard pH meter (METTLER TOLEDO, Shanghai, China), calibrated with pH 4.00 and 6.86 buffer solutions before measurement. Three independent samples were measured for each treatment and sampling time. Nitrite was determined using the spectrophotometric method in GB 5009.33-2016 [17]. Briefly, 5.0 g of homogenized sample was mixed with 12.5 mL of saturated borax solution, transferred quantitatively to a 500 mL volumetric flask with approximately 300 mL of water at 70 °C, and heated in a boiling-water bath for 15 min. After protein precipitation and clarification with potassium ferrocyanide and zinc acetate solutions, the extract was diluted to volume and filtered. An aliquot of the filtrate was reacted with sulfanilic acid and *N*-(1-naphthyl)ethylenediamine dihydrochloride, and the absorbance was measured at 538 nm. Nitrite concentration was calculated from a sodium nitrite standard curve and expressed as mg/kg.

For microbiological analysis, 25 g of pickle sample was aseptically transferred to a sterile homogenization bag containing 225 mL of sterile physiological saline and homogenized for 1–2 min to obtain a 10^−1^ suspension. Serial tenfold dilutions were then prepared. Total viable counts were determined according to GB 4789.2-2022 [18] by pour-plating 1 mL of appropriate dilutions in duplicate with 15–20 mL of plate count agar cooled to 46–50 °C, followed by incubation at 36 ± 1 °C for 48 ± 2 h. Lactic acid bacteria were enumerated according to GB 4789.35-2023 [19] by pour-plating 1 mL of appropriate dilutions in duplicate with MRS agar and incubating the plates anaerobically at 36 ± 1 °C for 48 h; incubation was extended to 72 h when colonies were absent or too small for reliable counting. Molds and yeasts were enumerated according to GB 4789.15-2016 [20] by plating 1 mL of appropriate dilutions in duplicate on rose bengal agar and incubating at 28 ± 1 °C, with the final count recorded on day 5. *Escherichia coli* counts were determined using the plate-count method according to GB 4789.38-2025 [21]. Appropriate serial dilutions were selected, and 1 mL of each dilution was inoculated into two sterile Petri dishes. Subsequently, 15–20 mL of tryptone bile X-glucuronide (TBX) agar, cooled to 48 ± 2 °C, was added to each plate and mixed thoroughly with the inoculum. After solidification, the plates were inverted and incubated at 36 ± 1 °C for 18–24 h. Blue-green colonies were considered typical *E. coli* colonies and enumerated. The results were expressed as log CFU/g. Microbial counts were expressed as log CFU/g. With a 10^−1^ initial suspension and 1 mL inoculation volume, the nominal detection limit of the plate-count procedures was 10 CFU/g. All microbiological determinations were performed using three independently prepared samples.

Sensory quality during storage was evaluated using the same trained 9-point sensory method described in Section 2.3.

The shelf life of the pickles was predicted using the Q10 model based on the ASLT methodology and the relevant method described in T/CNFIA 001-2017 [22]. The shelf-life endpoint was determined by integrating sensory rejection, microbial growth, and physicochemical changes. For two storage temperatures, *T*_1_ and *T*_2_, with corresponding shelf lives *θ*_S_(*T*_1_) and *θ*_S_(*T*_2_), the *Q*10 value was calculated as follows:

(1) $$Q_{10}={\left[\frac{\theta_{S}\left(T_{1}\right)}{\theta_{S}\left(T_{2}\right)}\right]}^{\frac{10}{T_{2}-T_{1}}}$$

The predicted shelf life at the target storage temperature was then calculated using:

(2) $$\theta_{S}\left(T\right)=\theta_{S}\left(T^{\prime }\right)\times Q_{10}^{\frac{T^{\prime }-T}{10}}\;$$

where *θ*_S_(*T*) is the predicted shelf life at the target temperature *T*, *θ*_S_(*T*′) is the observed shelf life at the accelerated temperature *T*′, and *T*′ − *T* is the temperature difference between the accelerated and target storage temperatures. In this study, the observed shelf lives at 30 °C and 40 °C were used to estimate *Q*10 and predict the theoretical shelf life at 4 °C.

### 2.5. Statistical Analysis

All experiments were performed in triplicate, and the data are expressed as the mean ± standard deviation. Because the number of repetitions was limited, differences among groups were evaluated using the non-parametric Kruskal–Wallis test, followed by Dunn’s multiple comparison test for post hoc pairwise comparisons. Statistical analyses were performed using IBM SPSS Statistics 17.0 (IBM Corp., Armonk, NY, USA). A value of *p* < 0.05 was considered statistically significant. Data visualization and graphing were conducted using Origin 2025b. Principal component analysis (PCA) and partial least squares-discriminant analysis (PLS-DA) of the flavor compounds were performed utilizing the MetaboAnalyst 6.0 online platform.

## 3. Results and Discussion

### 3.1. Heat Transfer Characteristics and Thermal Inactivation Kinetics

**Figure 1:**
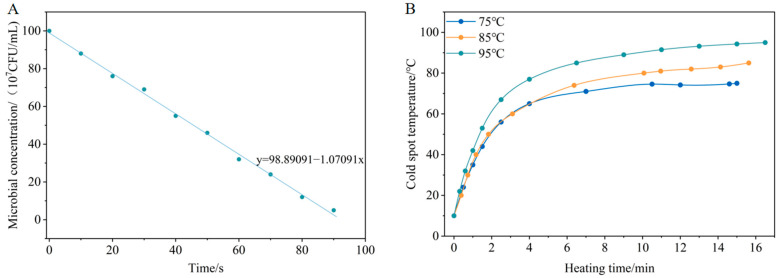
(**A**): Heat penetration curves of vacuum-packaged fermented pickled mustard greens; (**B**): Thermal inactivation curve for *Bacillus subtilis* at 85 °C.

Heat transfer characteristics are a crucial basis for determining the parameters of the heat treatment process, as they directly relate to the sterilization efficacy and energy consumption. In this study, 200 g of vacuum-packaged pickles were used, and temperature monitoring was conducted with the geometric center of the packaging bag serving as the cold spot. To accurately obtain the temperature change pattern of the cold spot, a temperature probe was placed at the geometric center of the vacuum bag to record the temperature changes in real-time under different water bath temperatures (75 °C, 85 °C, and 95 °C), and heat transfer curves were plotted. As shown in Figure 1A, the cold spot temperature exhibited a typical nonlinear increasing trend over time under different water bath temperatures. Specifically, in the 75 °C water bath, it took 15.0 min for the cold spot temperature to rise from the initial 15 °C to 75 °C; in the 85 °C water bath, it took 15.63 min to reach 85 °C; and in the 95 °C water bath, it took 16.5 min to reach 95 °C. This indicates that as the water bath temperature increases, the heating rate of the cold spot accelerates, but the time required to reach the target temperature does not shorten proportionally. This phenomenon is related to factors such as the inherent thermal conductivity of the pickle matrix, the thickness of the vacuum packaging bag, and the heat transfer boundary conditions. Although vacuum packaging eliminated the air inside the bag and reduced gas thermal resistance, pickles, as a solid–liquid mixed system, possess a low thermal conductivity, which still resulted in a heating lag in the central region.

The core objective of the pickle heat treatment process is to effectively inactivate heat-resistant microorganisms while maximizing the retention of its flavor and texture, thereby preventing spoilage during storage. Common microorganisms in fermented vegetables include lactic acid bacteria, yeasts, molds, enterobacteria, and *Bacillus* species [23]. Among them, *Bacillus* can form heat-resistant spores and is the most heat-resistant microbial group in the system [24]. Therefore, this study selected *Bacillus* as the target indicator bacterium for the design of the heat treatment process.

*Bacillus subtilis* preserved in the laboratory was selected as the indicator microorganism to establish heat treatment parameters by determining its thermal inactivation curve. The results, as shown in Figure 1B, indicate that the survival curve of *B. subtilis* spores at 85 °C exhibits a typical log-linear decline, conforming to first-order reaction kinetics. The calculated decimal reduction time (D-value) at this temperature—the time required to reduce the microbial population by one logarithmic cycle at 85 °C—was 1.37 min (Table 1). Further combining this with the Z-value (taken as 8 °C in this study, representing the temperature change required for a 10-fold change in the D-value), the D-values at 75 °C and 95 °C were calculated as 17.8 min and 3.36 s, respectively. These results demonstrate that the acidic environment significantly reduced the heat resistance of the spores. A 2D sterilization principle was adopted, which aims to reduce the target *Bacillus* spore count by 2 logarithmic cycles (99% lethality) to achieve commercial sterility and meet shelf-life requirements. Based on conservative considerations, three heat treatment schemes were designed: treatment at 75 °C for 64 min, at 85 °C for 19 min, and at 95 °C for 17 min.

### 3.2. Effects of Different Heat Treatment Conditions on Volatile Flavor Compounds

**Figure 2:**
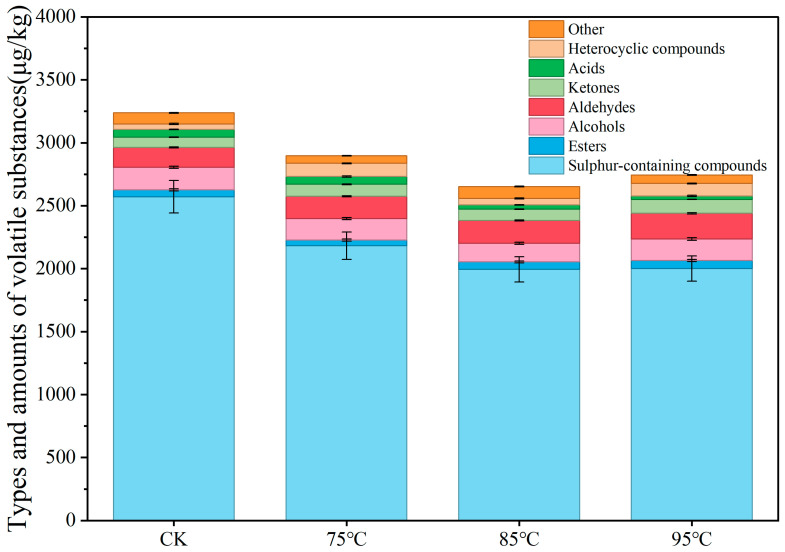
Types and semi-quantitative contents of volatile flavor compounds in fermented pickled mustard greens under different heat treatment conditions. Contents are expressed as μg 2-methyl-3-heptanone equivalents/kg sample, assuming a relative response factor of 1. Error bars indicate standard deviation.

The volatile flavor compounds of pickled mustard greens subjected to different heat treatments were detected using HS-SPME-GC-MS, identifying a total of 84 compounds, which were classified by chemical structure into sulfur-containing compounds, esters, alcohols, aldehydes, ketones, acids, heterocyclic compounds, and others. As shown in Figure 2, different heat treatment conditions altered the composition and semi-quantitative contents of volatile flavor compounds. Isothiocyanates are characteristic aroma-active compounds in mustard-derived fermented vegetables and are mainly responsible for the pungent, fresh, and sharp mustard-like aroma produced from glucosinolate hydrolysis. Therefore, mitigation of isothiocyanate degradation is important for retaining the typical sensory identity of fermented pickled mustard greens. The content of sulfur-containing compounds (primarily isothiocyanates) decreased after intensified heating, indicating that thermal treatment promoted volatilization, degradation, or conversion of these substances [12,14]. Simultaneously, heat treatment increased the contents of ketones, aldehydes, and heterocyclic compounds, which may be attributed to high-temperature-induced Maillard reactions and lipid oxidation [25].

**Figure 3:**
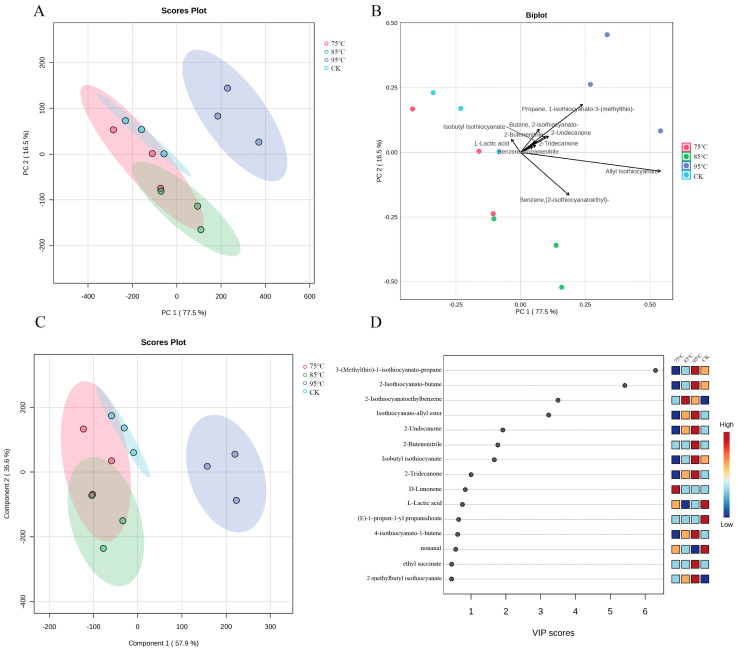
(**A**): PCA score plot based on GC-MS; (**B**): PCA biplot/loading plot based on GC-MS; (**C**): PLS-DA score plot based on GC-MS; (**D**): Key volatile compounds identified using the GC-MS combined with PLS-DA method.

To visually demonstrate the overall differences in volatile flavor compounds among pickles under different heat treatments, a principal component analysis (PCA) was performed on the volatile compounds of the three heat-treated groups (Figure 3A,B). The cumulative variance contribution rate of the first two principal components (PC1 and PC2) reached 94%, which adequately reflects the differences between the samples. The PCA score plot revealed a distinct separation of the four groups in the two-dimensional space. The control group and the 75 °C and 85 °C treatment groups were primarily located in the second and third quadrants, whereas the 95 °C treatment group was clearly separated from the other groups, locating in the first quadrant. This indicates that heat treatment temperature is the primary factor affecting the composition of volatile flavor compounds in pickles, with the 95 °C treatment causing the most substantial alteration to the flavor profile. The loading plot analysis indicated that PC1 was mainly contributed by isothiocyanates (e.g., phenethyl isothiocyanate, 3-methylthiopropyl isothiocyanate), ketones (e.g., 2-undecanone), and alcohols (e.g., 3,7,11,15-tetramethyl-2-hexadecen-1-ol). These compounds were highest in the 95 °C group, driving this group towards the positive direction of PC1. PC2 was mainly associated with acids, certain terpenes, and specific sulfur-containing compounds unique to the control group, separating the control and 75 °C groups from the high-temperature groups along the PC2 axis. The inter-group distances reflect the extent of the heat treatment’s impact on flavor compounds: the 75 °C and 85 °C groups were closer to the control group, suggesting that low-temperature treatments cause relatively mild flavor changes; the 95 °C group was the furthest from the others, demonstrating that high-temperature treatment markedly reshapes the volatile flavor composition of pickles.

To further identify the differential flavor markers under various heat treatments, a partial least squares-discriminant analysis (PLS-DA) was employed for supervised multivariate statistical analysis (Figure 3C,D). The PLS-DA score plot further validated the previous conclusion, showing a clear separation of the 95 °C group from the other three groups. Differential markers were screened based on variable importance in projection (VIP) values (VIP > 1). Among these, isothiocyanates were dominant; compounds such as phenethyl isothiocyanate, 3-methylthiopropyl isothiocyanate, and allyl isothiocyanate were the most critical markers for distinguishing the different treatment groups. These compounds were highest in the 95 °C group, imparting a strong spicy mustard flavor to the pickles, which is the core flavor characteristic of mustard pickles.

### 3.3. Sensory Evaluation of Pickles Under Different Heat Treatment Conditions

Sensory quality is a crucial indicator for evaluating the efficacy of the pickle heat treatment process. To comprehensively assess the effects of different heat treatments on the sensory characteristics of pickles, a trained sensory evaluation panel was organized to conduct a descriptive analysis and overall acceptability evaluation of the unsterilized control group and the three heat-treated groups, covering both flavor and quality dimensions.

**Figure 4:**
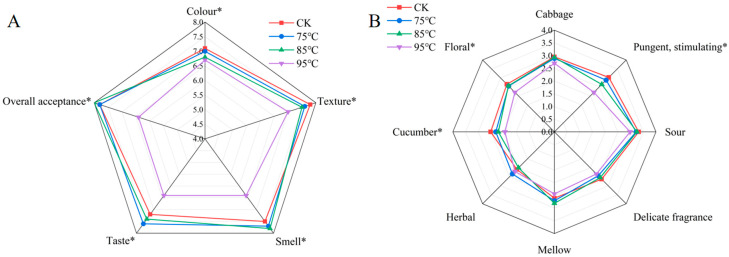
(**A**): Sensory quality radar chart for pickles under different heat treatment conditions. (**B**): Flavor profiles of pickles under different heat treatment conditions. Asterisks (*) indicate statistically significant differences among treatment groups (Kruskal–Wallis test followed by Dunn’s post hoc test, *p* < 0.05). Significant differences are indicated.

Figure 4A presents the sensory quality radar chart of pickles under different heat treatment conditions. From the overall morphological perspective, the unsterilized control group and the 75 °C and 85 °C treatment groups exhibited larger polygon areas with similar shapes, whereas the polygon for the 95 °C treatment group noticeably shrank, indicating that high-temperature treatment adversely affected multiple quality attributes of the pickles. Specifically, regarding color, the three heat-treated groups and the control group showed similar scores, suggesting that heat treatment has a minimal impact on pickle color. Texture is a core element of the pickle’s mouthfeel, mainly reflected in crispness and chewiness. The texture scores of the 75 °C and 85 °C groups were similar to the control group, but the 95 °C group scored lower than the other groups, indicating that high-temperature treatment may cause the degradation of cellulose, hemicellulose, and pectin in pickles, leading to texture softening and decreased crispness [26,27,28]. Regarding aroma and flavor, the 75 °C and 85 °C groups were slightly superior to the control group, while the 95 °C group scored lower. In terms of overall acceptability, the scores of the 75 °C and 85 °C groups were close to the control group, while the 95 °C group scored lower.

Figure 4B illustrates the flavor profile radar chart of pickles under different heat treatment conditions. From the overall flavor profile, the four groups of samples presented similar polygonal shapes across most flavor dimensions, indicating that the heat treatments did not fundamentally alter the flavor characteristics of the pickles, but differences existed in key flavor intensities. The spicy and pungent flavor is the core characteristic flavor of mustard pickles, primarily originating from isothiocyanate compounds. Its intensity weakened as the sterilization temperature increased, with the 95 °C treatment group exhibiting the lowest intensity. Refreshing and mellow aromas are important manifestations of pickle flavor coordination. The 85 °C treatment group scored higher in these aspects than the other heat-treated groups and was the closest to the control group. In background flavor dimensions such as herbal, cucumber, cabbage, and floral notes, the 95 °C group clearly deviated from the control group, whereas the other three groups were relatively close to each other.

Integrating the quality attribute evaluation and flavor profile analysis, it is evident that different heat treatment conditions have distinctly different impacts on the sensory quality of pickles. The 95 °C treatment group showed marked adverse effects across multiple sensory dimensions. While the scores of the 75 °C and 85 °C groups were close, the latter scored higher in flavor dimensions such as mellow and refreshing aromas. Furthermore, the excessively long processing time at 75 °C is not conducive to industrial application. Therefore, among the three evaluated conditions, 85 °C for 19 min was selected as the preferred heat treatment condition for pickles. This condition can achieve the expected microbial inactivation target while showing the highest observed retention of the color, texture, and characteristic flavor of the pickles, achieving a good balance between safety and sensory quality.

### 3.4. Quality Changes of Pickles During Accelerated Storage

#### 3.4.1. Changes in pH and Nitrite Content

**Figure 5:**
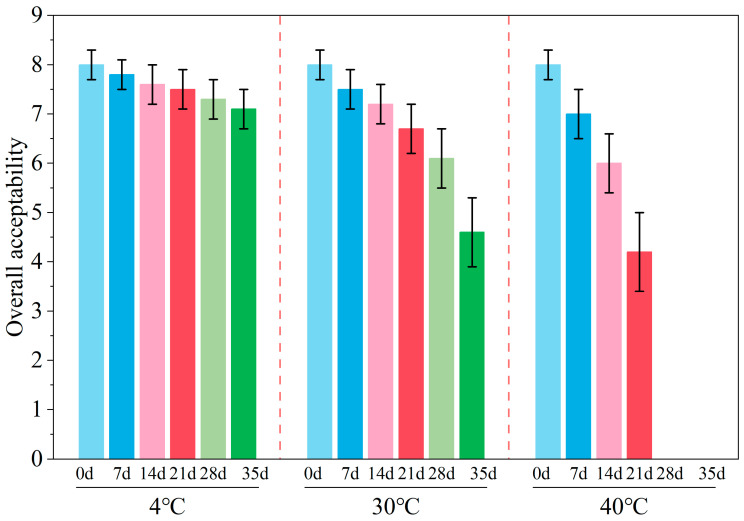
Changes in overall acceptability of fermented pickled mustard greens during accelerated storage.

The changes in the pH and nitrite content of the sterilized pickles during storage at different temperatures are presented in Table 2 and summarized graphically in Figure 5. As the storage time increased, the pH values of the pickles at all storage temperatures exhibited an overall slow declining trend, but the magnitude of the decline was closely related to the storage temperature. Under 40 °C storage conditions, the pickle pH dropped from an initial 3.28 to 3.08 within 21 days; at 30 °C, the pH decreased from 3.26 to 3.06 over 35 days; whereas at 4 °C, the pH only slightly decreased from 3.21 to 3.12 after 35 days, remaining almost stable. Kruskal–Wallis analysis indicated temperature-dependent pH changes during storage. These results demonstrate that low-temperature storage can effectively inhibit the post-acidification process of pickles and maintain pH stability. Because the pH of the pickles had already dropped below 4.0 after fermentation maturity, this restricted the further metabolic activity of microorganisms to a certain extent, resulting in a relatively small pH drop. This has positive implications for extending the shelf life of pickles, indicating that a low initial pH is a crucial guarantee for quality stability.

As shown in Table 2, the nitrite content of the pickles under different storage temperatures remained at a low level throughout the entire storage period without any marked variation trend. Across all temperature conditions, the nitrite content stabilized below 1 mg/kg, which is far below the national standard limit [11]. This indicates that the selected heat treatment effectively inhibited the growth and reproduction of microorganisms and reduced the degradation of nitrates, ensuring that there is no risk of nitrite accumulation in the pickles during storage. These findings are highly consistent with the recent study by S. Liu et al. [15], who analyzed the physicochemical characteristics and bacterial diversity of traditionally pickled mustard tubers from various regions in China. They also reported that under standard fermentation and storage conditions, nitrite residues are maintained at trace levels that strictly comply with food safety standards.

#### 3.4.2. Microbiological Changes

The changes in the total viable count, lactic acid bacteria (LAB) count, mold and yeast count, and *Escherichia coli* count of the pickles during storage at different temperatures are shown in Table 3. With the extension of storage time, the total viable count of pickles at all temperatures exhibited an upward trend, but the growth rate was highly dependent on the storage temperature. The initial total viable count of the pickles after heat treatment was 0.80 ± 0.12 lg CFU/g (approximately 6.3 CFU/g), which is far below the national standard limit for microbial total counts in pickled vegetables (104 CFU/g), indicating that the 85 °C/19 min heat treatment effectively reduced the microbial load. During storage, the growth rate of the total viable count differed across different temperatures. At 40 °C, the total viable count increased most rapidly, reaching 6.23 ± 0.18 lg CFU/g by day 21, exceeding the national standard limit (GB 2714-2015 [29]), indicating that residual microorganisms multiplied rapidly under high-temperature storage and product safety could not be guaranteed. At 30 °C, the growth rate was moderate, reaching 5.34 ± 0.22 lg CFU/g on day 35, which also exceeded the standard limit, suggesting a limited shelf life under room temperature. Conversely, at 4 °C, the total viable count increased very slowly, reaching only 1.78 ± 0.15 lg CFU/g by day 35, remaining well below the national standard limit throughout the period. This confirms that cold storage effectively inhibits the proliferation of residual microorganisms and is a key measure for maintaining the microbiological safety of pickles.

Throughout the monitored storage period, LAB were not detected (<10 CFU/g) under any temperature condition. This result demonstrates the strong lethal effect of the 85 °C/19 min heat treatment on LAB. As non-spore-forming bacteria, the heat resistance of LAB vegetative cells is far lower than that of *Bacillus* spores, and they can typically be effectively inactivated at 60–70 °C [30,31]. The 85 °C temperature used in this study far exceeded the lethal temperature for LAB.

Similarly, molds, yeasts, and *Escherichia coli* were not detected (<10 CFU/g) throughout the monitored storage period across all conditions. This can be attributed to several factors: firstly, molds and typical aerobic spoilage bacteria cannot grow and reproduce due to the lack of oxygen in the vacuum packaging; secondly, the low pH environment (pH < 4.2) of the pickles exerts a strong inhibitory effect on *Escherichia coli*, which generally cannot survive at a pH < 4.5 [32]; thirdly, the 85 °C/19 min heat treatment was sufficient to kill molds and *Escherichia coli*, and the vacuum packaging effectively isolated the product from external microbial contamination during storage. Furthermore, no bag swelling was observed during the entire storage period, further confirming the excellent microbiological stability of the product and the absence of massive proliferation by gas-producing microorganisms (e.g., yeasts, gas-producing *Bacillus*).

#### 3.4.3. Changes in Sensory Quality

Sensory quality is a key indicator for evaluating the shelf life of pickles, directly reflecting consumer acceptability. The changes in the sensory scores of pickles during storage at different temperatures are presented in Table 4. As storage time progressed, the sensory quality of the pickles exhibited temperature-dependent deterioration trends.

Under 4 °C storage, the sensory quality of the pickles remained relatively stable. The overall acceptability score only decreased slightly from the initial 8.0 ± 0.3 to 7.1 ± 0.4 over 35 days, while attributes such as color, texture, odor, and taste all remained at high levels. This result indicates that cold storage can effectively delay quality deterioration and maintain excellent sensory characteristics, providing a strong guarantee for extending the shelf life.

Under 30 °C storage, the rate of sensory quality deterioration accelerated noticeably. In the early stage of storage (0–21 days), the overall acceptability score dropped from 8.0 ± 0.3 to 6.7 ± 0.5, with all sensory indicators showing a steady decline. By day 28, the overall acceptability score fell to 6.1 ± 0.6, the texture score to 6.2 ± 0.6, and the odor and taste scores to 6.4 ± 0.6 and 6.0 ± 0.6, respectively. On day 35, the overall acceptability score further plummeted to 4.6 ± 0.7, with panelists reporting an obvious flavor imbalance, rendering the product unacceptable. This indicates that the shelf life of pickles at 30 °C is approximately 28 days, primarily limited by flavor imbalance and rapid sensory quality decline.

Under 40 °C storage, the sensory quality deteriorated the fastest. By day 7, all sensory indicators had declined, with the overall acceptability score dropping to 7.0 ± 0.5. By day 14, the overall acceptability score had fallen to 6.0 ± 0.6. On day 21, the overall acceptability score plummeted to 4.2 ± 0.8, and the scores for color, texture, odor, and taste all dropped below 5. The product exhibited obvious spoilage characteristics, including color browning, excessive softening, off-odors, and flavor deterioration. Sensory evaluation was discontinued after 21 days, determining that the shelf life at 40 °C does not exceed 14 days.

Synthesizing the sensory quality changes, it can be concluded that the sensory shelf life of pickles at 4 °C exceeds 35 days, maintaining good flavor, color, and texture. The shelf life at 30 °C is approximately 28 days, primarily limited by flavor deterioration. The shelf life at 40 °C is less than 14 days due to rapid spoilage.

### 3.5. Shelf-Life Prediction

To rapidly assess the shelf life of pickles treated at 85 °C/19 min under 4 °C refrigeration, an accelerated storage test (ASLT) combined with the Q10 method was utilized for prediction. Based on the ASLT results, integrating sensory evaluation, microbiological indicators, and physicochemical indicators, the actual shelf life of the pickles at different temperatures was determined as follows: 14 days at 40 °C and 28 days at 30 °C. Because the actual storage period at 4 °C exceeded 35 days, a predictive calculation was necessary. According to Equation (1), the Q10 value was calculated to be 2.0. Subsequently, using Equation (2), the predicted shelf life of the 85 °C/19 min sterilized pickles under 4 °C storage conditions was calculated to be 170 days. This predicted value is longer than the shelf lives at 30 °C (28 days) and 40 °C (14 days), demonstrating the advantage of low-temperature refrigeration in extending shelf life, which is consistent with the findings of Trindade et al. [33].

## 4. Conclusions

Targeting the bottlenecks of post-acidification, microbial contamination, and quality deterioration in fully fermented pickled mustard greens, this study evaluated three thermal processing conditions designed from heat penetration and microbial inactivation kinetics. By coupling the heat transfer characteristics of the pickle matrix with the thermal inactivation kinetics of *B. subtilis*, the study established treatments meeting a defined 2D microbial-reduction target. HS-SPME-GC-MS combined with multivariate statistical analysis showed that intensive heating at 95 °C caused substantial losses of characteristic sulfur-containing compounds, represented by isothiocyanates, and was associated with texture softening. Comparative sensory evaluation indicated that, among the three tested conditions, 85 °C for 19 min provided the best observed balance between the microbial-reduction target and retention of crisp texture and characteristic pungent and refreshing flavor. Combined with accelerated shelf-life testing and the Q10 model, the selected condition was predicted to provide a refrigerated shelf life of 170 days at 4 °C. These findings support the evidence-based selection of thermal processing conditions for industrial fermented vegetables; however, the study did not perform formal mathematical optimization over a continuous temperature-time design space.

## Figures and Tables

**Table 1:** Heat treatment conditions for pickles.

Temperature (°C)	D Value (min)	F_0_ (min)	Heat Transfer Time (min)	Total Heat Treatment Time (min)	Sterilization Time (min)
75	24.4	48.8	15	63.8	64
85	1.37	2.74	15.63	18.37	19
95	0.077	0.154	16.5	16.654	17

**Table 2:** pH and nitrite content of pickles under different storage temperatures. Values are expressed as mean ± SD. Different letters indicate significant differences at *p* < 0.05. Different lowercase letters within the same column indicate significant differences among storage times at the same temperature. ‘-’ indicates that samples stored at 40 °C were not analyzed after 21 days because obvious spoilage occurred and sampling was stopped.

Storage Time (d)	pH			Nitrite Content (mg/kg)		
	4 °C	30 °C	40 °C	4 °C	30 °C	40 °C
0	3.21 ± 0.01 ^c^	3.26 ± 0.01 ^d^	3.28 ± 0.01 ^d^	0.64 ± 0.06 ^ab^	0.64 ± 0.02 ^ab^	0.64 ± 0.01 ^b^
7	3.26 ± 0.02 ^d^	3.21 ± 0.01 ^c^	3.22 ± 0.01 ^c^	0.61 ± 0.05 ^ab^	0.68 ± 0.02 ^bc^	0.57 ± 0.02 ^a^
14	3.21 ± 0.01 ^c^	3.17 ± 0.02 ^b^	3.13 ± 0.01 ^b^	0.71 ± 0.02 ^b^	0.61 ± 0.06 ^ab^	0.78 ± 0.02 ^b^
21	3.22 ± 0.01 ^c^	3.15 ± 0.01 ^b^	3.08 ± 0.02 ^a^	0.57 ± 0.04 ^a^	0.55 ± 0.02 ^a^	0.65 ± 0.06 ^ab^
28	3.16 ± 0.02 ^b^	3.08 ± 0.01 ^a^	-	0.66 ± 0.05 ^ab^	0.71 ± 0.05 ^c^	-
35	3.12 ± 0.01 ^a^	3.06 ± 0.01 ^a^	-	0.59 ± 0.01 ^ab^	0.68 ± 0.05 ^c^	-

**Table 3:** Total viable count, LAB count, mold and yeast count, and *E. coli* count in pickles under different storage temperatures. Values are expressed as mean ± SD. Different letters indicate significant differences at *p* < 0.05. Different lowercase letters within the same column indicate significant differences among storage times at the same temperature. ‘-‘ indicates that samples stored at 40 °C were not analyzed after day 21 because spoilage had occurred. ‘ND’ indicates not detected (<10 CFU/g).

Count (log CFU/g)	Temperature/°C	Storage Time (d)					
		0	7	14	21	28	35
Total viable count (TVC)	4	0.80 ± 0.12 ^a^	0.84 ± 0.16 ^a^	0.99 ± 0.20 ^ab^	1.27 ± 0.16 ^bc^	1.49 ± 0.23 ^cd^	1.78 ± 0.15 ^d^
	30	0.80 ± 0.12 ^a^	1.34 ± 0.18 ^b^	2.28 ± 0.45 ^c^	3.12 ± 0.33 ^d^	4.27 ± 0.24 ^e^	5.34 ± 0.22 ^f^
	40	0.80 ± 0.12 ^a^	1.68 ± 0.16 ^b^	3.46 ± 0.66 ^c^	6.23 ± 0.18 ^d^	-	-
Lactic acid bacteria	4	ND	ND	ND	ND	ND	ND
	30	ND	ND	ND	ND	ND	ND
	40	ND	ND	ND	ND	ND	ND
Molds and yeasts	4	ND	ND	ND	ND	ND	ND
	30	ND	ND	ND	ND	ND	ND
	40	ND	ND	ND	ND	ND	ND
*Escherichia coli*	4	ND	ND	ND	ND	ND	ND
	30	ND	ND	ND	ND	ND	ND
	40	ND	ND	ND	ND	ND	ND

**Table 4:** Sensory evaluation scores for pickles under different storage temperature conditions. Values are expressed as mean ± SD. Different letters indicate significant differences at *p* < 0.05. Different lowercase letters within the same column indicate significant differences among storage times at the same temperature, and different uppercase letters within the same row indicate significant differences among temperatures at the same storage time.

Storage Time (d)	Storage Temperature (°C)	Color	Texture	Aroma	Flavor	Overall Acceptability
0	4	6.8 ± 0.3 ^Aa^	7.5 ± 0.2 ^Aa^	7.8 ± 0.3 ^Aa^	7.4 ± 0.3 ^Aa^	8.0 ± 0.3 ^Aa^
	30	6.8 ± 0.3 ^Aa^	7.5 ± 0.2 ^Aa^	7.8 ± 0.3 ^Aa^	7.4 ± 0.3 ^Aa^	8.0 ± 0.3 ^Aa^
	40	6.8 ± 0.3 ^Aa^	7.5 ± 0.2 ^Aa^	7.8 ± 0.3 ^Aa^	7.4 ± 0.3 ^Aa^	8.0 ± 0.3 ^Aa^
7	4	6.7 ± 0.3 ^Aa^	7.4 ± 0.3 ^Aa^	7.6 ± 0.3 ^Aa^	7.3 ± 0.3 ^Aa^	7.8 ± 0.3 ^Aa^
	30	6.5 ± 0.4 ^Aab^	7.2 ± 0.3 ^Aab^	7.4 ± 0.4 ^Aab^	7.1 ± 0.4 ^Aab^	7.5 ± 0.4 ^Aab^
	40	6.2 ± 0.5 ^Bb^	6.8 ± 0.4 ^Bb^	7.0 ± 0.5 ^Bb^	6.7 ± 0.5 ^Bb^	7.0 ± 0.5 ^Bb^
14	4	6.6 ± 0.3 ^Aab^	7.3 ± 0.3 ^Aa^	7.5 ± 0.3 ^Aa^	7.2 ± 0.3 ^Aa^	7.6 ± 0.4 ^Aab^
	30	6.3 ± 0.4 ^Abc^	6.9 ± 0.4 ^Bbc^	7.1 ± 0.4 ^Bbc^	6.8 ± 0.4 ^Bbc^	7.2 ± 0.4 ^Bbc^
	40	5.8 ± 0.5 ^Bbc^	6.2 ± 0.5 ^Cc^	6.3 ± 0.6 ^Cc^	6.0 ± 0.6 ^Cc^	6.0 ± 0.6 ^Cc^
21	4	6.5 ± 0.4 ^Aab^	7.2 ± 0.3 ^Aa^	7.4 ± 0.4 ^Aa^	7.1 ± 0.4 ^Aa^	7.5 ± 0.4 ^Aab^
	30	6.1 ± 0.5 ^Bcd^	6.6 ± 0.5 ^Bcd^	6.8 ± 0.5 ^Bcd^	6.5 ± 0.5 ^Bcd^	6.7 ± 0.5 ^Bcd^
	40	4.8 ± 0.7 ^Cc^	4.5 ± 0.8 ^Cd^	4.6 ± 0.8 ^Cd^	4.0 ± 0.8 ^Cd^	4.2 ± 0.8 ^Cd^
28	4	6.4 ± 0.4 ^Aab^	7.1 ± 0.4 ^Aa^	7.3 ± 0.4 ^Aa^	7.0 ± 0.4 ^Aa^	7.3 ± 0.4 ^Aab^
	30	5.8 ± 0.6 ^Bde^	6.2 ± 0.6 ^Bde^	6.4 ± 0.6 ^Bde^	6.0 ± 0.6 ^Bde^	6.1 ± 0.6 ^Bde^
	40	-	-	-	-	-
35	4	6.3 ± 0.4 ^Ab^	7.0 ± 0.4 ^Aa^	7.2 ± 0.4 ^Aa^	6.9 ± 0.4 ^Aa^	7.1 ± 0.4 ^Ab^
	30	4.9 ± 0.7 ^Be^	4.8 ± 0.7 ^Be^	5.0 ± 0.7 ^Be^	4.5 ± 0.7 ^Be^	4.6 ± 0.7 ^Be^
	40	-	-	-	-	-

## Data Availability

The raw data supporting the conclusions of this article will be made available by the authors on request.
